# Towards novel liver injury therapies based on design, synthesis and therapeutic efficacy of novel sulfone bis-compound on liver necrosis

**DOI:** 10.1038/s41598-025-02483-0

**Published:** 2025-05-20

**Authors:** Huda R. M. Rashdan, Sarah Hassan, Sara Maher, Hend Okasha

**Affiliations:** 1 Chemistry of Natural and Microbial Products Department, Pharmaceutical and Drug Industries Research Institute, National Research Centre, 33 El Buhouth St, Dokki, Giza, 12622 Egypt; 2 Electron Microscopy Department, Theodor Bilharz Research Institute, Giza, Egypt; 3 Immunology Department, Theodor Bilharz Research Institute, Giza, Egypt; 4 Biochemistry and Molecular Biology Department, Theodor Bilharz Research Institute, Giza, Egypt

**Keywords:** Sulfone bis-compound, TAA, Liver necrosis, Inflammation, Apoptosis, Biochemistry, Biotechnology, Chemical biology, Drug discovery

## Abstract

Liver necrosis is the irreversible loss of hepatocytes through toxin-induced injury, ischemia, or infection to produce organ dysfunction. It is a significant pathological marker in many liver disorders, including cirrhosis, and hepatitis, and contributes to organ failure and general systemic effects. This research aims to evaluate the protective effects of a newly synthesized compound named 1-(5-((1-(1-(4-((4-(4-(1-((5-acetyl-3-phenyl-1,3,4-thiadiazol-2(3H)-ylidene)hydrazono)ethyl)-5-methyl-1H-1,2,3-triazol-1-yl)phenyl)sulfonyl)phenyl)-5-methyl-1H-1,2,3-triazol-4-yl)ethylidene)hydrazono)-4-phenyl-4,5-dihydro-1,3,4-thiadiazol-2-yl)ethan-1-one (TTTE) sulfone-bis chalcone derivative on liver necrosis caused by TAA therapy using murine model. The research investigates optimal cellular pathways which demonstrate the therapeutic properties of TTTE as a potential treatment for liver injuries. The newly prepared compound TTTE was successfully characterized by Fourier transform infrared spectroscopy (FT-IR), proton nuclear magnetic resonance (1H-NMR) spectroscopy, carbon-13 nuclear magnetic resonance (13C-NMR), The safety of the as-prepared compound TTTE was determined based on weight changes and the behaviors in all the groups were monitored for 21 days. The effect of treatment of TTTE at different doses (300, 200, and 100 mg/kg B.W.) was studied. High-dose TTTE revealed a 62.5% survival rate compared to the untreated TAA group (40%). Molecular analysis exhibited that high-dose TTTE downregulated Cas-3, TIMP-1, and proinflammatory cytokines (TNF-α, NF-κB, and IL-6) compared to untreated TAA. Results of histopathological and IHC examinations exhibited high TTTE dose have no signs of liver injury with suppression in TGF-β expression as a result of anti-inflammatory response. Our study concluded that the synthesized compound, TTTE has a potential therapeutic strategy in mitigating liver necrosis.

## Introduction

**Fig. 1:**
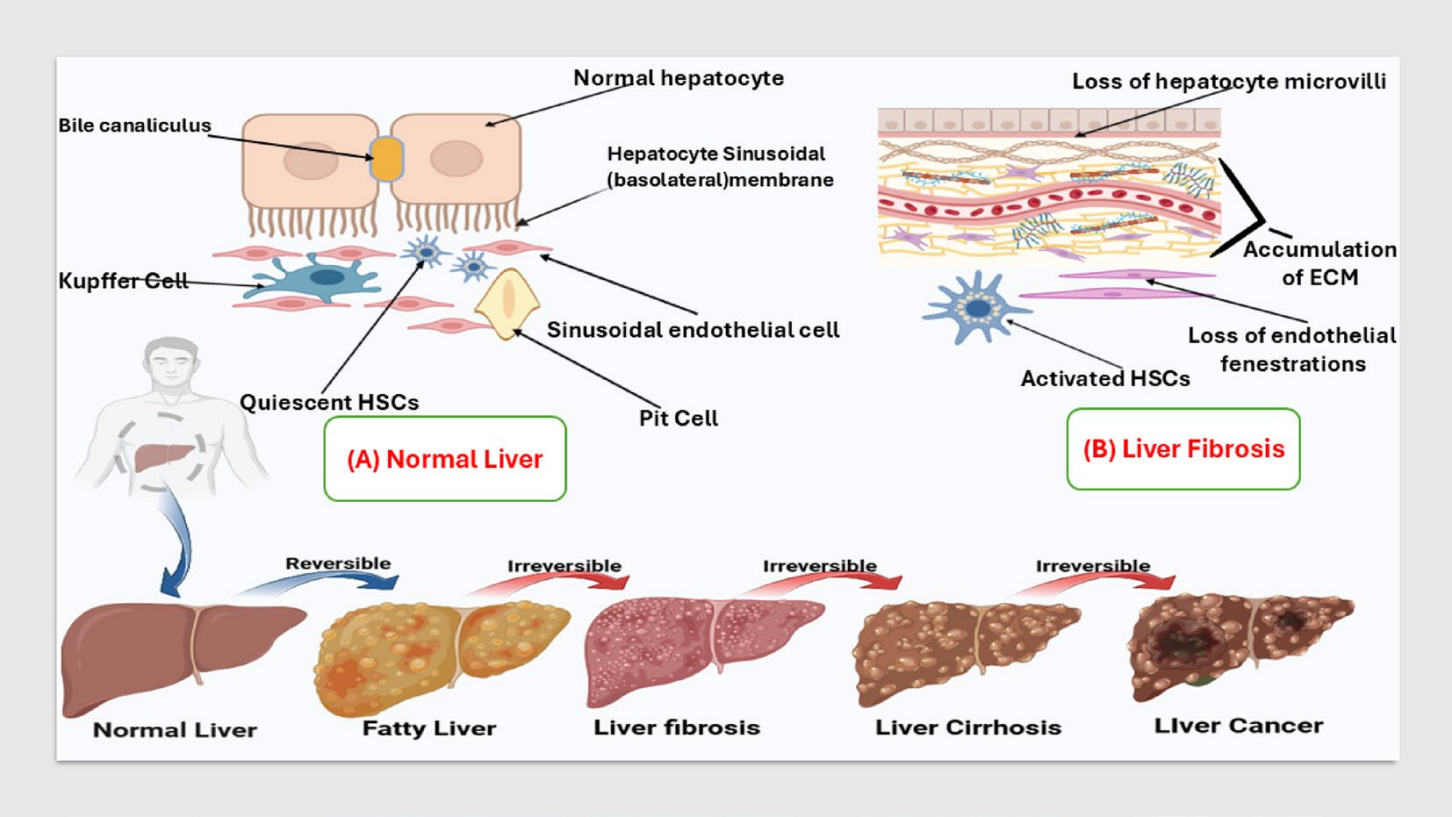
Normal and fibrotic liver architecture.

Liver diseases cause substantial worldwide health problems due to their annual toll of more than 2 million deaths. Cirrhosis-related complications alone cause 1 million deaths each year, while the other 1 million fatalities consist mainly of viral hepatitis and hepatocellular carcinoma^1^. The World Health Organization (WHO) recognizes chronic liver conditions such as viral hepatitis, alcoholic liver disease, nonalcoholic fatty liver disease (NAFLD), and cirrhosis as leading causes of global morbidity and mortality^2,3^. The Middle East experiences high rates of liver diseases because hepatitis B and C virus infections are widespread, while metabolic syndrome and NAFLD incidents continue to rise. Studies throughout the region indicate hepatitis C virus infection is the leading cause of chronic liver disease, cirrhosis, and hepatocellular carcinoma in numerous Middle Eastern nations^4^. These conditions are often associated with hepatic necrosis, a pathological process involving extensive inflammation or direct hepatocellular toxicity that can lead to progressive liver dysfunction and fibrosis (Fig. 1

Liver fibrosis can be considered a pathogenic case that leads to the loss of the architecture of the liver tissue and excessive accumulation of the extracellular matrix proteins (ECM). The resulting damaged liver tissues limit and alter the vital processes of the liver. This pathogenic case is caused by repeated and various insults to the liver likewise viral infections (chronic hepatitis B or C virus), parasitic or fungal infections, lipid accumulations, iron overload, chronic inflammations, genetical metabolic disorders, prolonged exposure to toxic agents, longterm and intense intake of some drugs (for instance; methyldopa, isoniazid, and chlorpromazine)^5–7^.

Moreover, liver necrosis may be caused by chronic alcoholism, viral hepatitis B and C, autoimmune diseases, drug toxicity (notably from acetaminophen overdose), and ischemic injury^8,9^. Managing liver necrosis is difficult and is based on identifying the exact cause of the disease^10–12^.

Liver necrosis activates the release of damage-associated molecular patterns (DAMPs) together with pro-inflammatory cytokines that include tumor necrosis factor-alpha (TNF-α), interleukin-1 beta (IL-1β), IL-6, and transforming growth factor-beta 1 (TGF-β1). These mediators play essential roles during hepatic stellate cell (HSC) activation. Kupffer cells and other immune cells produce these cytokines after DAMPs are released from necrotic hepatocytes, creating a pro-fibrogenic microenvironment. The potent fibrogenic protein TGF-β1 makes HSCs change from their quiescent state into an activated myofibroblast-like form, which shows augmented proliferation behavior and advanced migration abilities and generates excessive ECM. Remodeling of ECM and further HSC activation occur through the dual mechanisms of matrix metalloproteinases (MMPs) and reactive oxygen species (ROS) enzymes that develop during necrosis. HSCs become more susceptible to TGF-β1 after exposure to Toll-like receptor 4 (TLR4) signals, which boost fibrogenic processes. The combined inflammatory signals released during necrosis and cellular stresses lead to HSC activation, which sustains liver fibrosis^6,13,14^.

The prognosis for patients with liver necrosis remains limited, with mortality rates varying significantly depending on the cause and extent of liver damage^15^. Notably, the detection and intervention are crucial in improving outcomes, highlighting the need for continued research into effective therapies and preventive measures for this severe liver condition.

Currently, available treatments for liver necrosis are mainly supportive and focus on managing complications rather than reversing hepatocellular damage or fibrosis progression. Most pharmacological options offer limited efficacy in modulating the underlying apoptotic, inflammatory, and fibrotic pathways. Therefore, there is a critical unmet need for novel compounds that can target multiple aspects of liver injury, including oxidative stress, inflammation, apoptosis, and fibrosis.

Sulfone bis-compounds possess distinctive chemical and biological properties that make them promising candidates for treating liver necrosis. Sulfone serves as a stable moiety that binds to both enzymes as well as signaling proteins associated with oxidative stress and inflammation. Previous studies on polyphenolic tricyclic sulfones act as allosteric inhibitors of liver pyruvate kinase, causing a recorded protection against metabolic stress in liver cells, which concludes that sulfone-containing compounds can modulate liver-specific enzymes and pathways. Furthermore, sulfone bis-compounds may influence signaling pathways related to apoptosis and cellular repair, supporting hepatocyte survival and regeneration^16,17^.

In light of these properties, **TTTE**—a newly synthesized sulfone bis-chalcone derivative—was designed to address the limitations of current therapies by simultaneously targeting key molecular mechanisms involved in liver injury.

This work was planned to determine the effectiveness of sulfone bis-compound at various concentrations in the treatment of liver necrosis and to determine the capacity of this compound to reduce the extent of biochemical inflammatory markers, as well as the overall histological alterations in the liver tissue. This approach was designed to provide information on the potential of **TTTE** as a therapeutic agent in treating liver necrosis and its progression to other severe liver diseases.

## Materials and methods

### Chemistry

#### Raw materials

Methyl hydrazinecarbodithioate, and Dapsone were procured from Sigma-Aldrish. Some reagents and solvents were used in this study like DMF, Diisopropylethylamine, Absolute ethanol, isopropyl alcohol 99%, and acetic acid 99.9% were imported also from Sigma-Aldrish, Milwau-Kee, USA. All the solvents and chemicals were used without further purification.

#### Instrumentation

All melting points were uncorrected and measured using an electrothermal device. The IR spectra were recorded (KBr discs) using a Shimadzu FT-IR 8201 PC spectrophotometer. ^1^H- and ^13^C-NMR spectra were recorded in (CD3)2SO solutions on a BRUKER 500 FT-NMR system spectrometer, and chemical shifts are expressed in ppm units using TMS as an internal reference.

#### Synthesis

A mixture of −2-(1-(5-methyl-1-(4-((4-(5-methyl-4-(1-(2-((methylthio)carbonothioyl)hydrazono)ethyl)−1*H*−1,2,3-triazol-1-yl)phenyl)sulfonyl)phenyl)−4,5-dihydro-1*H*−1,2,3-triazol-4-yl)ethylidene)hydrazine-1-carbodithioate (**CPSC**) (**1**)^18,19^ (3.36 gm, 5 mmol) and N-(phenyl)−2-oxopropanehydrazonoyl chloride (**2**) (2.41 gm, 10 mmol) were heated under reflux in 20 mL ethanol containing catalytic amount of **DIPEA** (2–3 drops) for 5 h. The solid formed was separated and recrystallized from dioxane to give the desired compound.

1-(5-((1-(1-(4-((4-(4-(1-((5-acetyl-3-phenyl-1,3,4-thiadiazol-2(3*H*)-ylidene)hydrazono)ethyl)−5-methyl-1*H*−1,2,3-triazol-1-yl)phenyl)sulfonyl)phenyl)−5-methyl-1*H*−1,2,3-triazol-4-yl)ethylidene)hydrazono)−4-phenyl-4,5-dihydro-1,3,4-thiadiazol-2-yl)ethan-1-one (**TTTE**) (**3**) as yellow crystals. Yield: 72%; FT-IR (KBr, cm^−1^): *v* 1680 (C = O), 1615(C = N), 1575(C = C); ^1^H-NMR (DMSO-d_*6*_): *δ* 2.36 (s, 6 H, 2 CH_3_), 2.44(s, 6 H, 2 CH_3_), 3.25 (s, 6 H, 2 CH_3_), 7.19–8.20 (m, 18 H, ArH)ppm; ^13^C-NMR (100 MHz, DMSO-d_*6*_): *δ* 11.26 (2 CH3), 13.84 (2 CH3), 15.67 (2 CH3), 121.68, 126.35, 128.92, 129.27, 132.66, 133.70, 138.66, 138.57, 141.17, 142.14, 142.49 (Ar-Cs), 158.00 (C = N), 163.82 (C = O); MS m/z (%): 896 (M^+^).

### Biological evaluation

#### Acute toxicity study

Before we examined the biological activity of the **TTTE** compound, an acute toxicity study was carried out to determine the drug’s safety. Herein, nine groups were used, each one which was administered with a specific concentration of **TTTE**. *Swiss albino* male mice (6–8 weeks old, 22–26 g weight) were used for an acute toxicity study. After one week of acclimatization, two-fold serial dilution was performed on the **TTTE** starting from 2560 mg/kg body weight (B.W) to 10 mg/kg B.W intraperitoneally (i.p) to give nine groups, and each group contained three mice. Each group was administered the **TTTE** three times for one week, then the mice were monitored for any behavioral signs or mortality for another two weeks. Mice were sacrificed, and liver function tests, aspartate aminotransferase (AST), and alanine transaminase (ALT) (Sclavo Diagnostics International, Italy) were determined^20,21^.

#### Liver necrosis using thioacetamide (TAA)

*Swiss albino* mice with 6–8 weeks old and 22–25 g B.W were used. Mice (from the Animal House of TBRI) were randomly divided into three experimental groups and then the animals were housed for one week. Group-I; the normal control group (*n* = 8), mice received 0.9% w/v of saline i.p. Group-II; TAA control group (*n* = 10), TAA (Sigma Aldrich, Cat. No. 163678) at a dose of 200 mg/kg body weight (B.W) i.p twice per week for eight consecutive weeks to induce liver necrosis. Group-III; **TTTE** treatment group (*n* = 30), as in Group-II the mice were firstly administered with TAA i.p then they were further subdivided into three subgroups to receive treatment with **TTTE** for eight consecutive weeks: Group-IIIa (TAA + **TTTE** 300 mg/kg BW): Mice were treated with **TTTE** at a dose of 300 mg/kg BW three times per week i.p. Group-IIIb (TAA + **TTTE** 200 mg/kg BW): Mice were treated with **TTTE** at a dose of 200 mg/kg BW, three times per week i.p. Group-IIIc (TAA + **TTTE** 100 mg/kg BW): Mice were treated with **TTTE** at a dose of 100 mg/kg BW, three times per week i.p. light anesthesia using a ketamine-xylazine mixture (ketamine 80–100 mg/kg and xylazine 10–12.5 mg/kg), ensuring anesthesia lasts 20–30 min. After the experiment, animals will undergo an 8-hour fasting period before being weighed. Blood samples will be collected from the retro-orbital venous plexus under light anesthesia using a ketamine-xylazine mixture (ketamine 80–100 mg/kg and xylazine 10–12.5 mg/kg), ensuring anesthesia lasts 20–30 min^22–25^. Table 1.

**Table 1:** Experimental design of TAA-induced liver necrosis and TTTE treatment.

**Group**	**Treatment**	**Dose & Route**	**Frequency**	**Duration**	**n**
**Group-I**	Normal Control	0.9% saline (i.p.)	-	8 weeks	8
**Group-II**	TAA Control	TAA 200 mg/kg (i.p.)	2×/week	8 weeks	10
**Group-IIIa**	TAA + TTTE (High Dose)	TTTE 300 mg/kg (i.p.)	3×/week	8 weeks	10
**Group-IIIb**	TAA + TTTE (Mid Dose)	TTTE 200 mg/kg (i.p.)	3×/week	8 weeks	10
**Group-IIIc**	TAA + TTTE (Low Dose)	TTTE 100 mg/kg (i.p.)	3×/week	8 weeks	10

#### Molecular detection of liver injury-related markers

Using particularly designed primers, the relative expressions of tissue inhibitor metalloproteinase-1 (TIMP-1) and Cas-3 were evaluated. Extraction of total RNA from liver tissues was carried out using TRIzol reagent (Sigma Aldrich, USA). The RevertAid First Strand cDNA Synthesis Kit (Thermo Fisher, USA) was then used to synthesize cDNA. The primers specified in Table2 and the Maxima SYBR Green/ROX qPCR Master Mix (2X) (Thermo Fisher, USA) were used for the quantitative PCR (qPCR) studies. The cycling conditions were as follows: 15 min of initial denaturation at 95 °C, 40 cycles of denaturation at 95 °C for 20 s, and 1 min of annealing/extension at 55 °C. The formula 2^-ΔΔCt was used to calculate the relative quantification.

**Table 2:** Primer sequence of the determined genes.

Gene	Primer sequence	Accession No.	Reference
B-actin	GGGAATGGGTCAGAAGGACT	>NM_007393.5	[26]
	CTTCTCCATGTCGTCCCAGT		
CAS-3	CTACAGGGTTTCATCCAG	NM_007527.4	[27]
	CCAGTTCATCTCCAATTCG		
TIMP-1	GATATGCCCACAAGTCCCAGAACC	NM_011593.3	[28]
	GCACACCCCACAGCCAGCACTAT		

#### Assessment of Cas-3, TNF-α, NF-κB, and IL-6 levels

Tissue samples were cut into small pieces and then homogenized in 500 µL of phosphate-buffered saline (PBS, pH 7. 4) in the ice using a glass homogenizer. To enhance the disruption of cell membranes, ultrasonication was performed. The homogenates were centrifuged at 1500 g for 15 min to pellet the cell debris from the supernatant. The supernatant was processed for analysis immediately. The level of Cas-3 was measured using a competitive ELISA kit (MyBioSource, USA). Interleukin-6 (IL-6) was quantified using an ELISA kit (RayBiotech, USA). Nuclear Factor kappa B (NF-κB) was detected with an ELISA kit (MyBioSource, USA), while Tumor Necrosis Factor-alpha (TNF-α) was determined using an ELISA kit (Thermo Fisher Scientific, USA).

#### Histopathological and Immunohistochemical analyses

These analyses were performed in a blinded manner with no knowledge of group type. Samples of isolated livers were promptly fixed in buffered formalin 10%. The liver was exposed to routine paraffin block processing. Under a light microscope, sections were produced and stained with Hematoxylin and Eosin (H&E) to assess hepatic architecture and detect inflammation, dysplasia, and necrosis. A Zeiss Axio microscope was used to compare the liver histology of multiple groups. Images were produced using the attached digital Mrc5 Zeiss camera. An individual sign of liver cell injury was counted in each group in 10 high-power fields (/10 hpf)^29^. 

For immunohistochemical analysis, Sect. 5 μm thick were cut on positively charged glass slides, deparaffinized, hydrated, and then treated for antigen retrieval at a high pH (pH 8) using an automated immunostainer (Dako, Denmark). Monoclonal TGF-β antibody (Cat. No. NHP-AB250, Creative Biolabs, USA) was used as primary antibodies at dilution 1:100. Mouse IgG Fc binding protein conjugated to Horseradish Peroxidase (m-IgG Fc BP-HRP, sc-525409, Abcam UK) was utilized as the secondary antibody at dilution 1:300. Streptavidin–biotin–peroxidase complex and peroxidase-DAB (3,3’diaminobenzidine) (Santa Cruz Biotechnology, USA) detection method was performed according to the manufacturer’s instructions. In each run, positive and negative control slides were included, and a tissue section was processed as described but the primary antibody was omitted^30^.

#### Statistical analysis

The study’s data were shown as the mean ± SD. GraphPad Prism 8 (San Diego, CA, USA) was used for performing One-way, two-way ANOVA, or T-test. A *p*-value of less than 0.05 was considered statistically significant.

## Results

### Synthesis of 1-(5-((1-(1-(4-((4-(4-(1-((5-acetyl-3-phenyl-1,3,4-thiadiazol-2(3 H)-ylidene)hydrazono)ethyl)−5-methyl-1 H-1,2,3-triazol-1-yl)phenyl)sulfonyl)phenyl)−5-methyl-1 H-1,2,3-triazol-4-yl)ethylidene)hydrazono)−4-phenyl-4,5-dihydro-1,3,4-thiadiazol-2-yl)ethan-1-one (TTTE) (3)

**Fig. 2:**
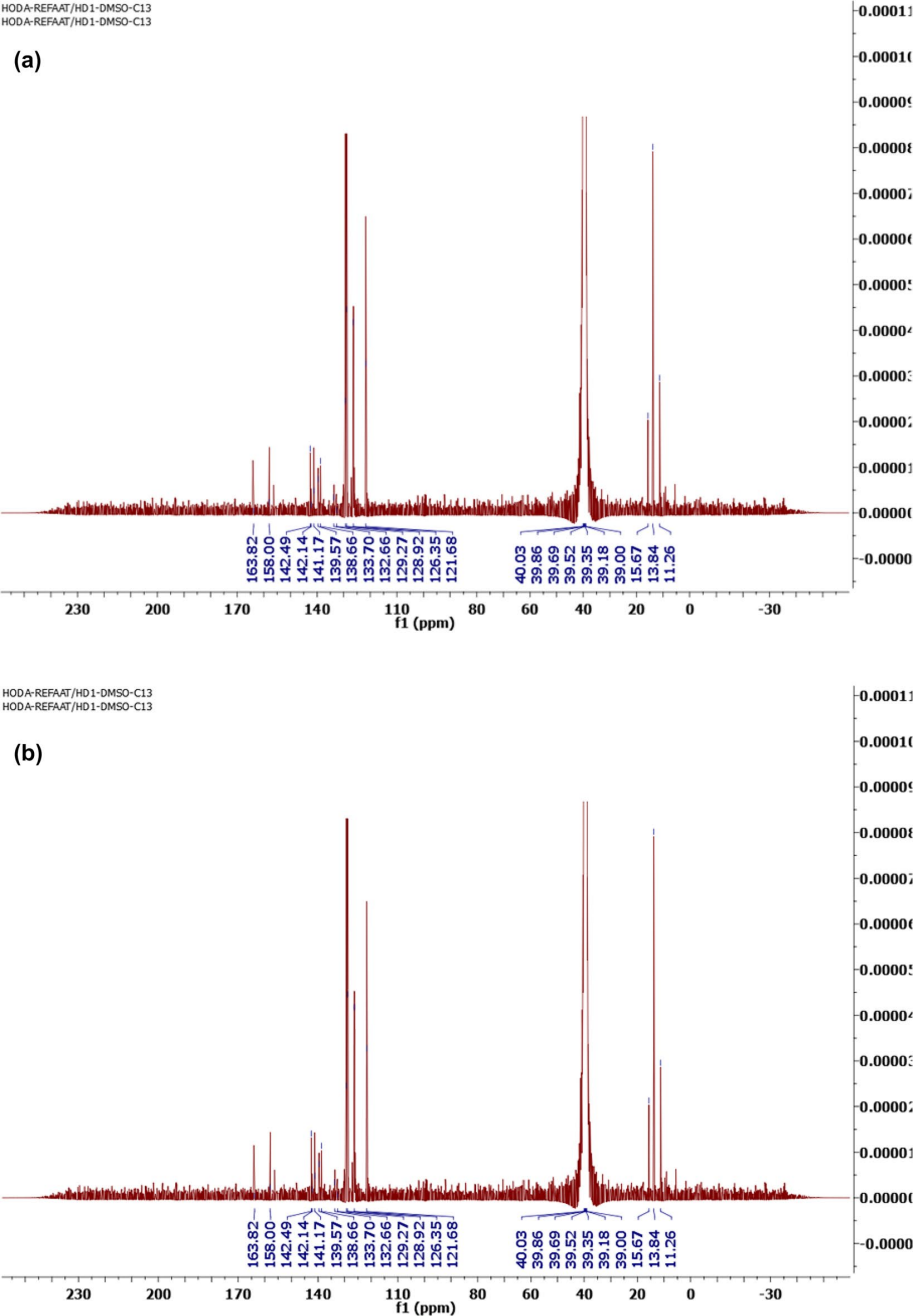
**A** H^1^NMR spectrum of 1-(5-((1-(1-(4-((4-(4-(1-((5-acetyl-3-phenyl-1,3,4-thiadiazol-2(3*H*)-ylidene)hydrazono)ethyl)−5-methyl-1*H*−1,2,3-triazol-1-yl)phenyl)sulfonyl)phenyl)−5-methyl-1*H*−1,2,3-triazol-4-yl)ethylidene)hydrazono)−4-phenyl-4,5-dihydro-1,3,4-thiadiazol-2-yl)ethan-1-one (TTTE) (3). **B** C^13^NMR spectrum of 1-(5-((1-(1-(4-((4-(4-(1-((5-acetyl-3-phenyl-1,3,4-thiadiazol-2(3*H*)-ylidene)hydrazono)ethyl)−5-methyl-1*H*−1,2,3-triazol-1-yl)phenyl)sulfonyl)phenyl)−5-methyl-1*H*−1,2,3-triazol-4-yl)ethylidene)hydrazono)−4-phenyl-4,5-dihydro-1,3,4-thiadiazol-2-yl)ethan-1-one (TTTE) (3).

**Scheme 1:**
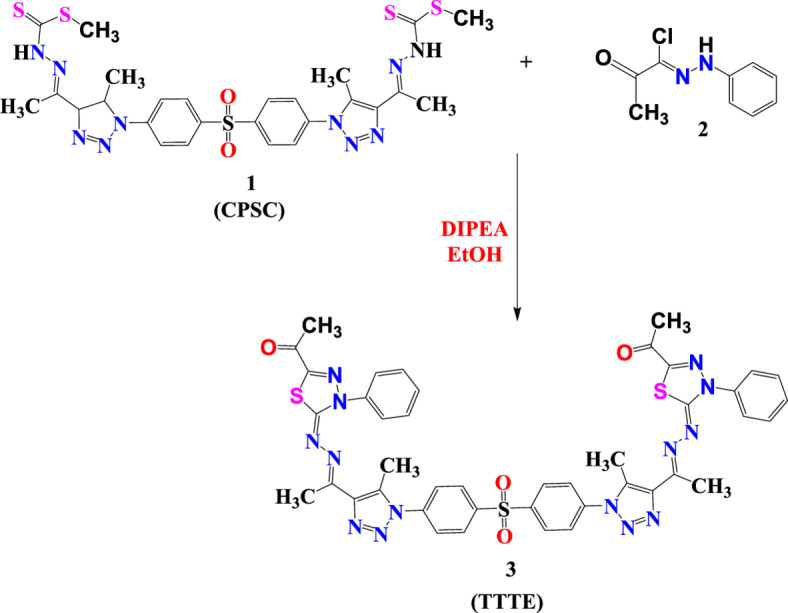
Synthetic procedures of 1-(5-((1-(1-(4-((4-(4-(1-((5-acetyl-3-phenyl-1,3,4-thiadiazol-2(3 H)-ylidene)hydrazono)ethyl)−5-methyl-1 H-1,2,3-triazol-1-yl)phenyl)sulfonyl)phenyl)−5-methyl-1 H-1,2,3-triazol-4-yl)ethylidene)hydrazono)−4-phenyl-4,5-dihydro-1,3,4-thiadiazol-2-yl)ethan-1-one (TTTE) (3)

2-(1-(5-methyl-1-(4-((4-(5-methyl-4-(1-(2-((methylthio)carbonothioyl)hydrazono)ethyl)−1*H*−1,2,3-triazol-1-yl)phenyl)sulfonyl)phenyl)−4,5-dihydro-1*H*−1,2,3-triazol-4-yl)ethylidene)hydrazine-1-carbodithioate (**CPSC**) (**1**) was submitted to react with N-(phenyl)−2-oxopropanehydrazonoyl chloride (**2**) in ethanol containing catalytic amount of DIPEA to afford the corresponding target molecules 1-(5-((1-(1-(4-((4-(4-(1-((5-acetyl-3-phenyl-1,3,4-thiadiazol-2(3*H*)-ylidene)hydrazono)ethyl)−5-methyl-1*H*−1,2,3-triazol-1-yl)phenyl)sulfonyl)phenyl)−5-methyl-1*H*−1,2,3-triazol-4-yl)ethylidene)hydrazono)−4-phenyl-4,5-dihydro-1,3,4-thiadiazol-2-yl)ethan-1-one (**TTTE**) (**3**) in a good yield (Scheme 1). The structure of **TTTE** was confirmed by spectral data, its H^1^NMR spectrum revealed three singlet signals at *δ* 2.36, 2.44, and 3.25 ppm attributed to the protons of the sex methyl groups. Additionally, the 18 aromatic protons appeared as multiplet signals in the range from *δ* 7.19 to *δ* 8.20 ppm (Fig. 2a). Moreover, its C^13^ NMR exhibited significant signals at 11.26, 13.84 and 15.67 ppm represented the sex methyl groups carbons and characteristic signals at 121.68, 126.35, 128.92, 129.27, 132.66, 133.70, 138.66, 138.57, 141.17, 142.14, 142.49 for the aromatic carbons, 158.00 for C = N and 163.82 for the C = O (Fig. 2b).

### Acute toxicity study

**Fig. 3:**
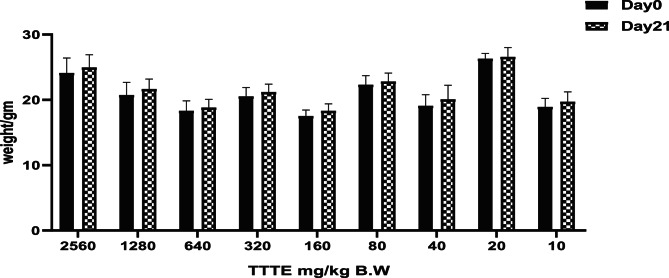
Body weight monitoring of mice in different TTTE dosage groups over three weeks of acute toxicity study.

Regarding the acute toxicity study of **TTTE**, the body weights of the mice in different groups were recorded on day zero and day twenty-one. The highest dose of 2560 mg/kg B. W. was given to the group which had an initial weight of 24.13 ± 2.27 gm on Day 0, which slightly increased to 24.97 ± 1.95 by Day 21. In addition, the group that was administered with the lowest dose of 20 mg/kg B. W. had the least variation with a weight of 18.93 ± 1.3 on Day 0 and 19.7 ± 1.54 gm on Day 21. In general, the groups experienced slight changes in body weight over the 21 days, and no significant weight loss at any of the doses was recorded. No behavioral changes of toxicity were noticed in any of the groups receiving different concentrations of **TTTE**. The mice were active, did not appear stressed, groomed, and fed normally. Further, there were no changes in the body posture or movement pattern. The absence of any behavioral abnormalities in all experimental groups suggested that **TTTE** administration did not induce any obvious neurobehavioral toxicity. Fig. 3.

Another confirmation of **TTTE** safety was liver function tests which revealed that after three weeks of the acute toxicity study, the liver enzyme values were in the normal range in all experimental groups. Table 3.

**Table 3:** Liver function tests (ALT/AST) on different acute toxicity groups.

IU/L	1	2	3	4	5	6	7	8	9
ALT	42.86±10.04	30.89±12.44	20.68±29.79	27.14±7.99	31.30±9.86	22.34±6.85	25.57±8.68	30.47±14.91	42.66±9.29
AST	79.85±21.96	63.56±10.91	68.44±16.07	72.41±24.94	70.49±21.00	58.95±27.63	57.79±15.95	62.79±12.72	84.85±13.87

### Liver necrosis using thioacetamide (TAA)

#### Physical parameters

**Fig. 4:**
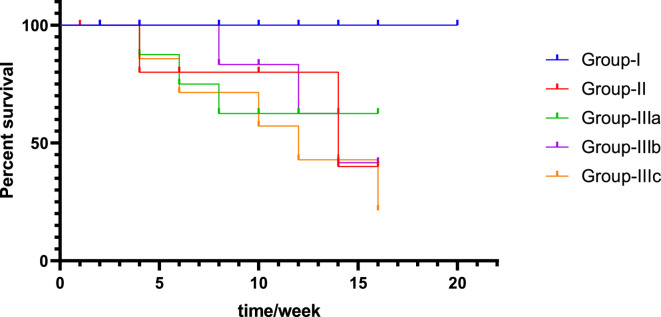
The graph illustrated here is Kaplan-Meier’s survival plot, which explains the probability of survival with time measured in weeks and various groups. On the X-axis, time is measured in weeks; on the Y-axis, the percentage of the survivors is given.

During the experimental study, the death was monitored between groups. Results showed that no mortality was observed in the normal control group (Group-I) with a 100% survival rate. The survival rate for Group-II started to drop at week 4 with a survival rate equal to 40%. Group-IIIa which was treated with a high concentration of **TTTE** had a survival rate of 62.5%. However, Group-IIIb and Group-IIIc had survival rates of 40% and 30%, respectively. Fig. 4.

#### Molecular detection of liver injury-related markers

**Fig. 5:**
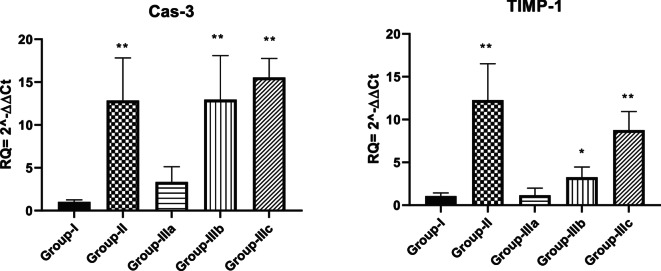
This bar graph represents the relative expression levels of Cas-3 and TIMP-1 genes. The groups represent different conditions: Group-I (normal control), Group-II (liver necrosis), Group-IIIa (necrosis treated with a high dose of TTTE), Group-IIIb (necrosis treated with a moderate dose of TTTE), and Group-IIIc (necrosis treated with a low dose of TTTE). Data are expressed as mean ± SEM, with *p < 0.05, **p < 0.01, ***p < 0.001, and ****p < 0.0001 compared to Group-I.

Group-I (normal control) showed a very low expression of Cas-3 and TIMP-1, as expected for a healthy, untreated liver. Group-II (liver necrosis) exhibited a remarked increase in the expression level of Cas-3 with a significantly elevated level of TIMP-1 which indicates a response to tissue injury and attempts at repair or fibrosis in the necrotic liver. Group-IIIa (treated with a high dose of **TTTE**) showed a substantial reduction in the expression of both TIMP-1 and Cas-3 compared to Group-II. This suggested that the high dose of **TTTE** possibly had limited tissue damage in the liver, indicating strong therapeutic properties. Group-IIIb (treated with a moderate dose of **TTTE**) and Group-IIIc (treated with a low dose of **TTTE**) also showed observed upregulation of Cas-3 and TIMP-1 compared to Group-IIIa. Fig. 5.

#### Assessment of Cas-3, TNF-α, NF-κB, and IL-6 Levels

**Fig. 6:**
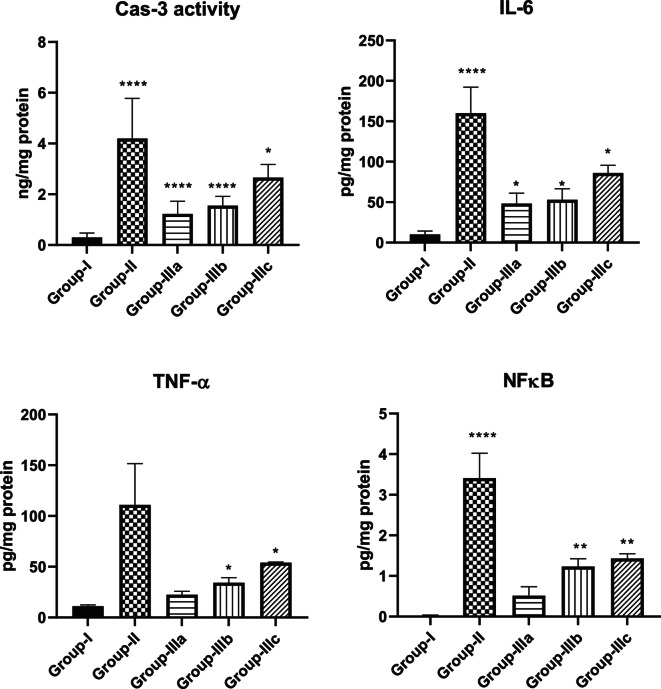
Effect of TTTE treatment on biochemical markers in liver necrosis (Caspase-3 activity, IL-6, TNF-α, and NF-κB levels). Data are expressed as mean ± SEM, with *p < 0.05, **p < 0.01, ***p < 0.001, and ****p < 0.0001 compared to Group-I.

Our findings indicated the change in biochemical parameters related to liver necrosis and the **TTTE** impact with different concentrations (Fig. 6). Group-II, which is the liver necrosis group, has a significantly higher Cas-3 activity, IL-6, TNF-α as well as the NF-κB levels compared to the normal group (Group-I) suggesting that apoptosis, inflammation, and activation of the NF-κB pathway. After **TTTE** treatment, all three groups IIIa, IIIb, and IIIc showed a decrease in these markers in a dose-dependent manner. High-dose **TTTE** treatment (Group-IIIa) significantly affects caspase-3 activity, IL-6, TNF-α, and NF-κB levels near the normal group showing the protective and anti-inflammatory effect. Group-IIIb and IIIc also exhibited a decrease in these markers, though lesser than Group-IIIa showing that **TTTE** had a therapeutic effect on liver necrosis although not as potent as the high dose.

#### Histopathological Examination

**Fig. 7:**
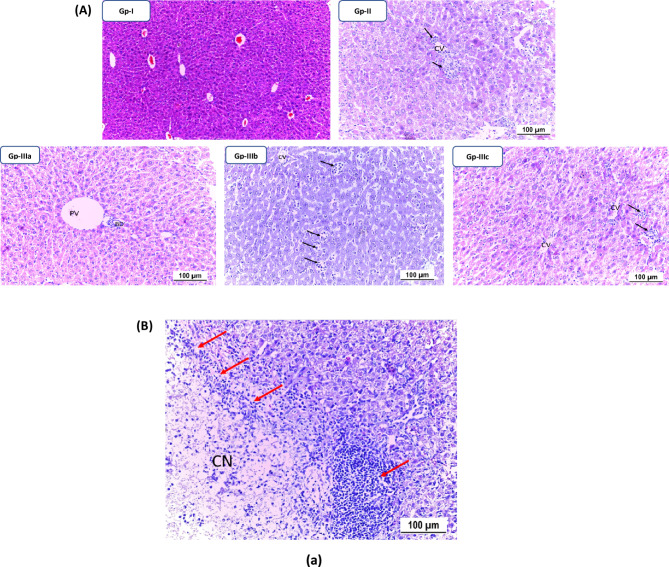

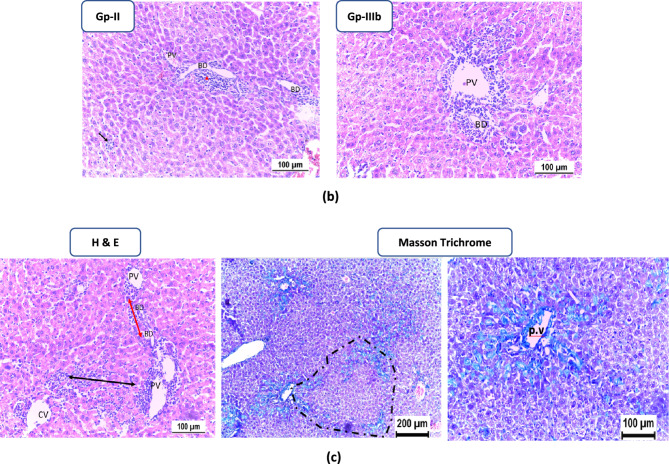
**A** Spotty necrosis detection (H & E stain) of mice liver tissue of different groups at low magnification power; spotty necrosis (black arrows); CV: central vein, PV: portal vein, BD: bile duct. Both normal and treated with high-dose TTTE groups (Group-I and Group-IIIa) showed no signs of liver cell injury with normal portal triad in addition to appearing hepatocytes with preserved configuration. TAA group (Group-II), moderate-dose and low-dose TTTE groups; Group-IIIb & Group-IIIc showed multiple spotty necrosis. **B **H & E stain of mice liver tissue; PV: portal vein, BD: Bile duct, CV: Central vein. (a) Confluent necrosis (CN) and surrounding lymphocytic infiltrate (red arrows) in low-dose TTTE group (Group-IIIc). (b): Mild interface hepatitis (red arrowhead) was observed in TAA group (Group-II) with spotty necrosis (black arrow) and moderate interface hepatitis was observed in moderate-dose TTTE group (Group-IIIb). (c): H & E stain of low-dose TTTE group (Group-IIIc) showed portal-portal bridging hepatitis (double head red arrow) and portal-central bridging hepatitis (double head black arrow). Masson trichrome staining showed evolving portal–portal bridging fibrosis with early nodular formation (dotted) in addition to evolving fibrous tissue deposited in sinusoid around hepatocytes.

In the TAA-treated group, liver injury was characterized by spotty necrosis and mild to moderate interface hepatitis with a few foci of bridging portal–portal inflammation. However, no confluent necrosis, bridging inflammation, or fibrosis was observed in this group. Group-IIIa, treated with 300 mg of **TTTE**, exhibited no signs of hepatocyte injury or fibrosis. Group-IIIb, treated with 200 mg of **TTTE**, showed multiple spotty necrosis, focal confluent necrosis, and moderate to severe interface hepatitis. Additionally, there were few foci of bridging inflammation (portal–portal, and portal–central) and minimal periportal fibrosis, as well as fibrosis surrounding necrotic nodules. In Group-IIIc, treated with the lowest **TTTE** dose (100 mg), multiple spotty necrosis was observed without confluent necrosis. However, mild to moderate interface hepatitis, frequent foci of bridging inflammation (portal–portal, and portal–central), and frequent foci of bridging fibrosis were present, though no frank cirrhotic nodules were detected. (Fig. 7a and b)

Spotty necrosis score (/10 hpf) showed that both TAA and the lowest **TTTE** dose groups had the highest spotty necrosis scores; 6.17 ± 2 and 7.17 ± 3.3, respectively. Group-IIIa treated with 300 mg of **TTTE** had the lowest spotty necrosis score (0.67 ± 0.5) with statistical significance correlated to Group-II, *p*-value = 0.0045.

#### Immunohistochemical analysis

Results of immunohistochemistry showed that both the moderated and low-dose **TTTE**-treated group (Group-IIIb and Group-IIIc) in addition to the TAA group had an obvious.

**Fig. 8:**
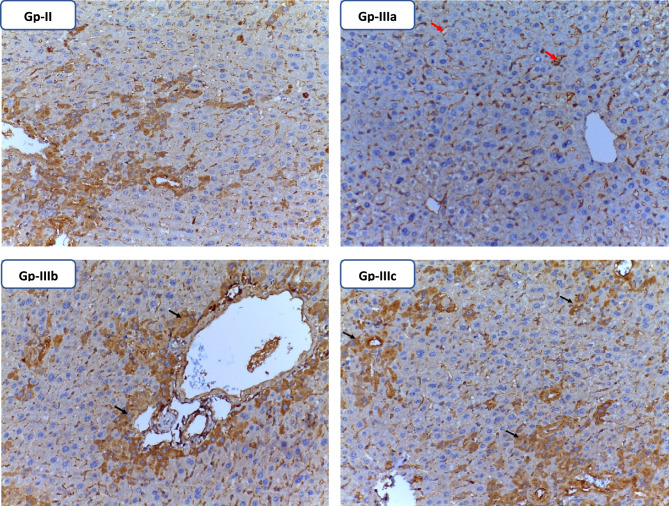
Immunohistochemical analysis of TGF-β expression in liver tissue stained with TGF-β antibody. In the TAA group (Gp-II), Gp-IIIb, and Gp-IIIc showed scattered cytoplasmic positivity in hepatocytes (black arrows). The high TTTE-treated group; Gp-IIIa, showed hepatocytes with a negative TGF-β immunohistochemical stain. Red arrows revealed non-specific immunohistochemical staining in the liver sinusoidal. (x20).

TGF-β expression which indicates an active inflammatory response. However, no expression of TGF-β was observed in the high-dose **TTTE**-treated group (Group-IIIa). These data are compatible with H & E in addition to Massone trichrome stains. Fig. 8.

## Discussion

In this study, we evaluated the potential therapeutic efficacy of **TTTE** in the treatment of liver necrosis induced by thioacetamide (TAA) in mice model. Our findings showed that B.W., in addition to the survival rate in the TAA group, remarkably decreased (40%). A 62.5% was shown in the high-dose **TTTE**-treated group, indicating the potential therapeutic efficacy of **TTTE** at this concentration (300 mg/Kg B.W). The moderate-dose (40%) and low-dose (30%) were considerably lower, which proved that lower concentrations of **TTTE** lack the potency to increase the survival percentage or to reduce the damaging effects observed in the TAA group. Like other chalcone hepatoprotectants, our derivative required higher concentrations (40%) to significantly improve survival, while lower doses (30%) showed negligible effects. Unlike classical chalcones^31^, the bis-sulfone moiety in TTTE may alter its dose-response profile, explaining the observed threshold effect^32,33^. Caspase-3 (Cas-3) is one of the most important enzymes involved in liver necrosis, especially in cases of apoptosis-mediated cell death. In liver necrosis, the activation of caspase-3 is essential in the apoptotic signaling cascade since it affects the liver’s capacity to regenerate^34,35^. Herein, the **TTTE** compound reduced the expression of genes associated with apoptosis; Cas-3, and TIMP-1 in a dose-dependent µmanner, showing that higher doses had an impact on liver injury treatment by reducing cell death and fibrogenesis. While the present study focused on transcriptional profiling of TIMP-1, we acknowledge that mRNA expression may not always reflect actual protein levels due to post-transcriptional regulation. Nevertheless, the observed mRNA expression patterns are consistent with established responses in liver injury models, where TIMP-1 upregulation is associated with fibrogenesis and tissue remodeling. The significant downregulation of TIMP-1 mRNA in TTTE-treated groups, particularly at the high dose, indicates a probable attenuation of fibrotic activity^36,37^. The results displayed a dosage-dependent decrease in essential inflammatory mediators TNF-α and NF-κB after TTTE treatment. TNF-α serves as a known apoptosis trigger of hepatocytes^38^, in addition, the activation of NF-κB sustains inflammatory cascades in liver injury, thus, their suppression potentially leads to decreased liver necrosis^39^. Our results showed a significant reduction in the levels of TGF-β, which stands as a central cytokine during fibrogenesis processes. TGF-β drives the development of liver fibrosis when it activates hepatic stellate cells (HSCs) that produce and deposit excessive ECM and collagen^40^. The downregulation of these critical profibrotic and pro-inflammatory markers suggests that TTTE exerts its hepatoprotective effects through multimodal mechanisms, targeting both necroinflammation and fibrotic progression. In our study, immunohistochemistry analysis on liver tissue treated with a low dose of the **TTTE** revealed the presence of TGF-β expression, indicating fibrosis activation. Also, H & E in addition to Masson trichrome revealed the same results by showing multiple spotty necrosis with moderate interface hepatitis, frequent foci of bridging inflammation (portal–portal, and portal–central), and frequent foci of bridging fibrosis. This suggests that the low dose may not be sufficient to fully inhibit liver injury; in tissues treated with moderate and high doses of the **TTTE**, no TGF-β expression was observed. These results suggest a mitigation of fibrogenic activity, although this reduction may not be sufficient to fully reverse established fibrosis, but likely contributes to slowing or halting the progression of fibrotic remodeling by attenuating HSC activation and ECM deposition^41^. These results recommended that high-dose of **TTTE** potentially reduce inflammation via downregulation of TNF-α and NF-κB, which play critical roles in hepatocyte apoptosis and chronic liver inflammation, in alignment with our experimental findings, which could accelerate fibrogenic processes.

### Conclusion

A study examined the hepatoprotective effect of TTTE, which is a modern sulfone-bis chalcone derivative, through laboratory tests on thioacetamide (TAA)-induced liver necrosis mouse subjects. The protection offered by TTTE depends on the dosage because it leads to better survival results and lessened tissue damage with a substantial decrease in the markers for cellular death and inflammation, such as Caspase-3, TNF-α, and NF-κB. The high dosage treatment with TTTE blocked fibrogenic signals since no TGF-β was detected in liver tissue alongside decreased fibrosis. These findings highlight the therapeutic relevance of chalcone-based compounds, particularly sulfone-bis chalcone derivatives like TTTE, in managing liver injury and fibrosis. The anti-apoptotic, anti-inflammatory, and anti-fibrotic properties observed in this study support TTTE’s potential as a promising candidate for further preclinical development. As interest in chalcone scaffolds continues to grow due to their diverse biological activities, this compound adds valuable insight into their possible application in liver disease therapy.

### Limitations and future perspectives

The study has various limitations, even with its promising results. We noticed that higher doses correlated with reduced TIMP-1 and Caspase-3 mRNA expression but we failed to obtain protein data at the same time. Fundamental research utilizing Western blotting and related analytical methods like ELISA and immunofluorescence must be done to verify these transcriptional modifications at the protein expression level. The relevance of using TAA-induced liver injury models in mice remains unclear when aiming to understand hepatotoxicity and fibrosis patterns in human patients because additional research into chronic liver models and clinical sample analysis is mandatory. The observations of survival rates in addition to the sample sizes need larger numbers of test subjects particularly within the low- and moderate-dose groups to establish statistical validity. Functional assessments measuring liver health such as ALT, AST liver enzyme levels, and bilirubin content in blood and oxidative stress markers should be added to future evaluations of TTTE due to their omission from the current investigation. Future studies on TTTE are required to determine its pharmacokinetic data, long-term safety, and molecular mechanism of action in detail. Expanding the assessment of TTTE’s therapeutic value includes tests for its performance in models of NASH and viral hepatitis conditions. Further research should explore whether combining TTTE with present-day hepatoprotective agents would generate combined therapeutic results that would promote innovative treatment method development. Translation studies, along with clinical trials, represent the essential basis for developing TTTE into a new therapeutic agent that might treat chronic liver diseases in people.

## Data Availability

all the data used are included in the manuscript
